# Content of selected heavy metals in the umbilical cord blood and anthropometric data of mothers and newborns in Poland: preliminary data

**DOI:** 10.1038/s41598-023-41249-4

**Published:** 2023-08-28

**Authors:** Joanna Grzesik-Gąsior, Jan Sawicki, Agnieszka Pieczykolan, Agnieszka Bień

**Affiliations:** 1 State University of Applied Sciences in Krosno, 38-400 Krosno, Poland; 2 Department of Analytical Chemistry, Medical University of Lublin, 20-059 Lublin, Poland; 3 Department of Coordinated Maternity Care, Faculty of Health Sciences, Medical University of Lublin, 20-059 Lublin, Poland

**Keywords:** Public health, Medical research

## Abstract

The ability to accumulate metals in organs and tissues leads to disturbances in the physiological functioning of the body, causing oxidative stress. This negatively affects the functioning of the placenta and may result in miscarriages, premature birth and fetal growth disorders. The aim of the study was to examine the relationship between the levels of selected heavy metals in umbilical cord blood and anthropometric parameters of mothers and the newborns. Content of elements in umbilical cord blood has been assessed by high-resolution inductively coupled plasma optical emission spectroscopy (ICP-OES). The study results were collected and statistically analyzed using IBM SPSS Statistics software (PS IMAGO). The Pearson correlation coefficient was used to test for associations between selected variables. Regression analysis was conducted to identify predictors of anthropometric parameters of studied women and newborns. The study group consisted of women aged 19–41, whose pregnancy was uncomplicated and were not exposed to heavy metals due to their work or smoking. The following metals were identified in all collected cord blood samples: lead (26.25 ± 9.32 µg/L), zinc (2025.24 ± 717.83 µg/L), copper (749.85 ± 203.86 µg/L), manganese (32.55 ± 13.58 µg/L), chromium (8.34 ± 2.16 µg/L) and selenium (158.46 ± 41.58 µg/L). The conducted statistical analysis indicated the relationship between the copper content in the umbilical cord blood and the weight gain of pregnant women. A significant relationship was observed between newborn head circumference and chromium content. In addition, significant positive correlations were found between the content of zinc and copper, manganese and lead, manganese and selenium, lead and selenium, and lead and chromium in umbilical cord blood. The ratio of zinc to copper concentrations was related to neonatal head circumference. Weight gain in pregnant women is positively correlated with the copper level in umbilical cord blood. There is an association between head circumference at birth and the chromium concentration in umbilical cord blood. Copper and zinc levels in umbilical cord blood are positively correlated with head circumference at birth.

## Introduction

Environmental levels of heavy metals vary considerably and may depend on the type of metal, its chemical form, proximity of metropolitan agglomerations or busy roads and even on the types of plants found in a given area. Heavy metal levels in human tissues also depend on the individual’s lifestyle choices, e.g. smoking. The accumulation of heavy metals in organs such as the brain, heart, liver and kidneys impairs the physical functioning of the body, damaging metabolic processes and causing oxidative stress^1^. Environmental risk factors that affect pregnancy and childbirth exert their effects not only during pregnancy but also during preconception. Heavy metals can affect both male and female reproductive functions. They can disrupt the menstrual cycle, lead to difficulties conceiving and can even cause birth defects^2–4^. Environmental pollution can have an impact on sperm quality^5,6^. Scientific research indicates that environmental pollution can have adverse effects not only on human fertility but also on pregnancy and can lead to pregnancy complications and negative pregnancy outcomes^6,7^. The placenta, which serves as a protective barrier for the fetus against toxins, is permeable to heavy metals. The redox imbalance caused by heavy metals has adverse effects on placental function. This may result in pregnancy failure (e.g., miscarriage), premature birth or fetal growth restriction^6,7^. The concentration of heavy metals in umbilical cord blood reflects the actual amount of the metals that crosses the placental barrier and reaches the fetus^8^. The exposure of pregnant women to heavy metals results in the accumulation of the metals in the umbilical cord blood and may have an impact on anthropometric parameters in newborns. Given its ability to readily cross the placenta, lead may adversely affect the development of the fetus and subsequently of the newborn. Accumulated in bones, it can be released into the mother’s bloodstream and then reach the fetus. Lead has a neurotoxic effect and disturbs the neurological development of the fetus. Furthermore, fetal hemoglobin has greater affinity to lead than adult hemoglobin. The female body is more susceptible to the effects of cadmium than the male body. Through its effect on hormonal pathways and oxidative stress generation, cadmium can play a role in pregnancy loss and other obstetric failures^7^. There have also been reports on the relationship between the prenatal exposure to cadmium and low birth weight, birth length and head circumference^9^. Exposure to chromium during pregnancy may be associated with fetal growth restriction. Higher chromium concentration in biological samples from pregnant women is associated with a smaller abdominal circumference and lower fetal weight, as assessed with ultrasound^10,11^. Trace elements such as zinc, copper and selenium play an important role in normal fetal growth, hence their adequate levels in umbilical cord blood are crucial for fetal development. Furthermore, these elements are positively correlated with birth weight in newborns^12^. Other studies indicate that copper or manganese levels are significantly associated with lower body weight in newborns^13,14^. Manganese is an important antioxidant in pregnancy, but its cord blood concentration, both low and high, may affect anthropometric birth parameters^15^. Analyses of the impact of heavy metals such as nickel, thallium or antimony on anthropometric parameters in newborns have shown that despite their low levels in cord blood, these metals have a negative effect on fetal growth^16^. The biological materials most commonly used to assess levels of exposure to environmental pollutants are blood, which is used to assess the levels of relatively persistent compounds, i.e. those with a half-life of several months or years, and urine, which is used to assess the levels of non-persistent compounds, i.e. those whose half-life is measured in hours^17^.‬

The aim of the present study was to examine the relationship between the levels of selected heavy metals in umbilical cord blood and anthropometric parameters of mothers and their newborns. The trace elements analyzed in the study are constituents of PM10 and PM2.5 particulate matter, as well as soil and drinking water contaminants. Exposure to the toxic effects of heavy metals in the preconception and prenatal period is an important marker of child and adult health around the world and in this context environmental pollution becomes an ever growing concern.

## Methods

### The aim and design of the study

The study was carried out among 134 women giving birth in hospitals in Lublin, Poland. The women were residents of Lublin Province, which is located in the east of Poland, near the country’s border with Belarus and Ukraine. The eastern border of the region forms part of the eastern border of the European Union. The key climate issue in the region are emissions from individual home heating systems and road transport. Elevated levels of particulate matter (PM10 and PM2.5) in the air are mainly recorded in cold months, when pollution levels from low-emission sources are higher. The environmental levels of the heavy metals monitored in the region are relatively low, below the target and limit values. Lublin Province ranks in the middle of Polish regions in terms of its pollution levels^18,19^. The study was carried out at the John of God Independent Public Regional Hospital in Lublin and the Cardinal Stefan Wyszynski Regional Specialized Hospital in Lublin. Research was suspended from November to December 2020 due to introduction of lockdown in Poland, related to the emergence of SARS-CoV-2, for this reason it is difficult to calculate the minimum number of people in the sample.

The umbilical cord blood samples analyzed in the study were collected between 2020 and 2021. The study was carried out once consent had been given by the heads of the delivery rooms where the study was conducted.

All women who consented to participate in the study and met the inclusion criteria took part in the study. The criteria for inclusion in the study were as follows: place of residence—Lublin County, age 18 or over, single, uncomplicated pregnancy, delivery at ≥ 38 gestational weeks, patient consent. The exclusion criteria were as follows: tobacco use, use of psychoactive substances, diagnosis of cancer or other disease complicating pregnancy, long-term occupational exposure to heavy metals, multiple pregnancy, maternal age under 18 years. A total of 200 women in labor were initially recruited for the study. However, as some of the women did not meet all the inclusion criteria, 134 blood samples were ultimately analyzed in the study.

### Assessments

The anthropometric data of mothers and newborns included in the study was collected by means of a diagnostic survey using a standardized questionnaire. The following data relating to the women studied was collected: age, body weight before pregnancy and pregnancy weight gain. The information collected relating to the newborns included the following anthropometric parameters: birth weight (in grams), birth length (in centimeters), head circumference and chest circumference at birth (in centimeters).

The head circumference is measured with a centimeter tape in the fronto-occipital dimension at the level of the frontal tuberosity and the occipital protuberance. The head circumference of a eutrophic newborn is 34–35 cm. The measurement of the chest circumference is carried out on the front wall of the chest at the level of the nipples, on the back at the line of the lower angles of the shoulder blades. The chest circumference of a eutrophic newborn is 32–33 cm.

Measurement of the newborn's body length begins with the measurement of the SI parietal-neck length—from the posterior (small) parietal along the spine to the beginning of the gluteal sulcus. Then the centimeter should be carried along the lower limb to the knee flexion and further to the newborn's heel. The length of the body is between 46 and 54 cm.

All the data relating to the women included in the study was provided by them in the questionnaire. Anthropometric measurements of newborns were made by the researchers themselves in the first hours of the newborn's life (the same measurement results were written in the medical documentation of newborns: Newborn's health history and Baby's health book).

### Element analysis

Blood was collected from the umbilical vessels, after the umbilical cord had been cut, into metal-free tubes with ethylenediaminetetraacetic acid (EDTA) disodium salt dihydrate. Each sample was labelled with the patient’s code number, as specified in the questionnaire and study participation consent form. The samples were then stored at − 25 °C until chemical analysis was performed. Digestion of blood sample was carried out using DigiPREP (SCP Science) heating block. After removal from the refrigerator, samples were left until they reached paper temperature. Then 2 mL of blood was drawn into DigiTUBE, 1.8 mL of 65% HNO3 Suprapur and 3.4 mL deionized water were added. Digestion was carried out in closed vessels at temperature 120 °C for 90 min. Digested samples were filtered and quantitatively transferred to Digitube vessels and filled up with deionized water to 10 mL.

The levels of heavy metals in the umbilical cord blood samples were measured by high-resolution inductively coupled plasma optical emission spectroscopy (ICP-OES) (PlasmaQuant PQ 9000 Elite, Analytik Jena). The correctness of the method was determined based on certified matrix reference materials. Gas flow were set as follows: nebulizer gas 0.6 L/min, auxiliary gas 0.5 L/min, plasma gas 14.0 L/min. The following analytical lines were used for analysis: Se—196,0280 nm, Zn, 206,2000 nm, Cu 213,5981 nm, Pb 220,3534 nm, Cd 228,8018 nm, Mn 257,6100 nm, Cr 267,7160 nm and the signal reading time was 3 s. Axial reading mode used for all elements, with the exception of manganese, which was determined using attenuated axial mode. The sample transport rate was set at 1.0 mL/min. Concentration measurements for each sample were made in triplicate. The accuracy of the method was determined based on certified reference materials (Seronom Whole Blood L2&L3). The values of the limits of quantification for the analyzed elements in the samples after digestion were as follows: Zn, Se, Pb, Cu—5 μg/L, Cr, Mn—1 μg/L.

The following metals were initially selected for analysis: lead, cadmium, chromium, nickel, zinc, copper, molybdenum, manganese, cobalt, antimony, thallium, vanadium and selenium. The following metals were found in all the umbilical cord blood samples collected: lead, zinc, copper, manganese and selenium. The levels of other metals (cadmium, nickel, molybdenum, cobalt, antimony, thallium and vanadium) were below the limit of detection (LOD).

**Figure 1:**
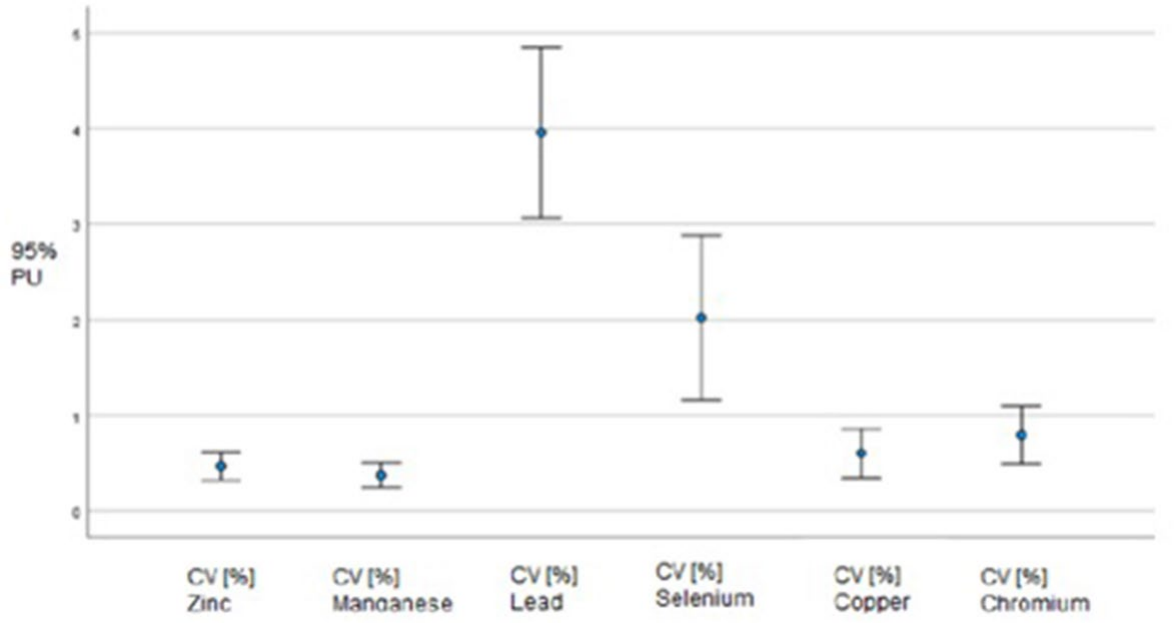
Variability of heavy metal levels in umbilical cord blood in relation to the mean − CV [%].

The precision of the measurements of heavy metal levels in umbilical cord blood was evaluated by the percentage coefficient of variance (CV%). The lowest level of precision was found for the measurements of lead concentration, whereas the highest level of precision was found for zinc and manganese, which was due to the levels of the metals in umbilical cord blood. The results of the measurements for 95% confidence intervals (95% CI) are shown in Fig. 1.

The study was carried out in accordance with the principles of the Declaration of Helsinki and the study design was approved by the Bioethics Committee of the Medical University of Lublin (KE-0254/280/2018).

### Statistical analysis

The study results were collected and statistically analyzed using IBM SPSS Statistics software (PS IMAGO). The values of measurable parameters analyzed in the study were presented using the mean (M), standard deviation (SD), median (me), minimum (Min) and maximum (Max) values. The Pearson correlation coefficient was used to test for associations between selected variables. Regression analysis was conducted to identify predictors of antropometric parameters of studied women and newborns. Regression analysis with moderating factors was performed to evaluate the variables (F-test). The level of statistical significance was set at *p* < 0.05.

### Ethics approval and consent to participate

The study was carried out in accordance with the principles of the Declaration of Helsinki and the study design was approved by the Bioethics Committee of the Medical University of Lublin (KE-0254/280/2018). Informed consent was obtained from all study participants. All methods will be carried out in accordance with relevant guidelines and regulations.

## Results

### Respondents’ characteristics and results of chemical analysis of umbilical cord blood samples

The mean age of the women was 30.07 ± 4.29 years, pregnancy weight gain experienced by women was 4.00–43.00 kg, with the mean of 14.34 ± 6.73 kg. Their body weight before pregnancy was 78.78 ± 10.81 kg. The mean birth weight of the newborns studied was 3501.49 ± 442.68 g. Their body length was 50–58 cm, with the mean of 54.10 ± 2.07 cm. The mean head circumference was 34.06 ± 1.35 cm and the mean chest circumference was 33.87 ± 1.58 cm.

Table 1 presented the mean concentrations of heavy metals. The zinc concentration in the samples reached the highest level when compared to the other metals analyzed and was 1165–4592 µg/L. The mean manganese concentration was 32.55 ± 13.58 µg/L. The mean lead level in umbilical cord blood was 26.25 ± 9.32 µg/L, with the highest concentration being 49.45 µg/L. The mean copper level was 749.85 ± 203.86 µg/L. Chromium levels ranged from 4.99 µg/L to 14.73 µg/L and were the lowest among all the elements analyzed.

**Table 1:** Results of heavy metal concentrations in umbilical cord blood.

Elements [µg/L]	M ± SD	Me (Min–Max)
Zinc (Zn)	2025.24 ± 717.83	1812.00 (1165.00–4592.00)
Copper (Cu)	749.85 ± 203.86	713.00 (393.60–1502.00)
Selenium (Se)	158.46 ± 41.58	156.00 (79.70–398.60)
Manganese (Mn)	32.55 ± 13.58	31.43 (9.80–73.10)
Lead (Pb)	26.25 ± 9.32	25.60 (2.76–49.45)
Chromium (Cr)	8.34 ± 2.16	8.45 (4.99–14.73)

*M* mean, *SD* standard deviation, *Me* median.

### Analysis of correlations between heavy metal concentrations in umbilical cord blood and anthropometric data from mothers and newborns

Table 2 presents correlations between heavy metal concentrations in umbilical cord blood and socio-demographic and anthropometric data from mothers and newborns. The statistical analysis did not find any statistically significant differences between the content of heavy metals in umbilical cord blood and the age of the respondents (p > 0.05).

**Table 2:** Correlations between heavy metal concentrations in umbilical cord blood and socio-demographic data from mothers and anthropometric data of newborns.

Parameters			Zinc (Zn)	Copper (Cu)	Selenium (Se)	Manganese (Mn)	Lead (Pb)	Chromium (Cr)
Maternal data	Age	r	− 0.135	− 0.17	− 0.013	0.007	− 0.121	0.3
		p	0.276	0.17	0.914	0.955	0.329	0.186
	Body weight before pregnancy	r	− 0.029	− 0.007	0.137	0.099	0.089	− 0.215
		p	0.818	0.957	0.269	0.424	0.472	0.349
	Weight gain during pregnancy	r	0.231	0.278	0.017	− 0.069	0.037	− 0.098
		p	0.06	0.023	0.894	0.582	0.766	0.673
Newborn data	Birth weight	r	0.023	0.096	0.122	0.078	0.271	− 0.295
		p	0.856	0.438	0.324	0.528	0.047	0.195
	Birth length	r	− 0.108	− 0.097	0.166	0.055	0.16	0.013
		p	0.385	0.436	0.18	0.661	0.197	0.955
	Head circumference	r	− 0.027	0.105	0.111	0.115	0.137	− 0.499
		p	0.828	0.399	0.370	0.352	0.270	0.021
	Chest circumference	r	− 0.14	− 0.15	0.087	0.115	0.189	− 0.264
		p	0.26	0.226	0.482	0.356	0.126	0.248

*r* Pearson correlation coefficient, *p* probability.

The analysis of correlations between the content of heavy metals in umbilical cord blood and body weight before pregnancy did not reveal any statistically significant differences (p > 0.05). However, there was a weak positive correlation between the mean copper concentration in umbilical cord blood and weight gain in pregnancy (p = 0.023).

A positive correlation was observed between the lead concentration and birth weight (p = 0.047). Furthermore, it was found that the chromium concentration in umbilical cord blood depends on the head circumference of the newborns – the greater the circumference, the lower the chromium level (p = 0.021). No correlation was found between the body length and chest circumference of the newborns and the content of heavy metals in umbilical cord blood (p > 0.05).

The univariate regression analysis performed for three pairs of variables was presented in Table 3. In the first case, the explained variable was the gestational weight gain of the women who took part in the study, and the explanatory variable was the copper content of cord blood. Based on the regression coefficients, it can be concluded that the concentration of copper in cord blood is positively related to pregnancy weight gain (β = 0.278; p < 0.05). The F-test result was statistically significant, and the model tested explains 7.7% of the variation in the dependent variable.

**Table 3:** Regression analysis for results for gestational weight gain, birth weight and neonatal head circumference.

Predictors	*B*	*SE*	*β*	*t*	*p*
	Weight gain during pregnancy F = 5.459; *p* = 0.022; R^2^ = 0.077				
(Constant)	7.455	3.053		2.441	0.017
Cu [µg/L]	0.009	0.003	0.278	2.336	0.022
	Birth weight F = 3.014; *p* = 0.087; R^2^ = 0.210				
(Constant)	3239.132	160.221		20.216	0.000
Pb [µg/L]	9.995	5.756	0.211	1.736	0.0872
	Head circumference F = 6.306; *p* = 0.021; R^2^ = 0.499				
(Constant)	36.575	0.940		38.901	0.000
Cr [µg/L]	− 0.274	0.109	− 0.499	− 2.511	0.021

*Cu* copper, *Pb* lead, *Cr* chromium, *β* standardized coefficients, *SE* bootstrapped standard errors.

In the second case, the explanatory variable was the newborn's birth weight, and the explanatory variable was the lead content in cord blood. Regression analysis showed that the higher the lead content in cord blood, the higher the birth weight of the newborn (β = 0.211; p > 0.05). The model tested explained 21.0% of the variance, but the F-test result was not statistically significant.

In the third case, a regression analysis was performed in which the explained variable was newborn’s head circumference, and the explanatory variable was chromium concentration in cord blood. The higher the chromium concentration in cord blood, the smaller newborn’s head circumference (β = − 0.499; p < 0.05). The model tested explained 49.9% of the dependent variable, and the F-test result was statistically significant (F = 6.306; p < 0.05).

The results of the statistical analysis demonstrated significant positive correlations between the content of zinc and copper, manganese and lead, manganese and selenium, lead and selenium, and lead and chromium. The correlation values ranged from 0.447 to 0.842 (p < 0.001)—Table 4.

**Table 4:** Correlations between heavy metal concentrations in umbilical cord blood.

	Zn	Cu	Se	Mn	Pb	Cr
Zn	–					
Cu	0.842*	–				
Se	0.082	0.103	–			
Mn	− 0.132	− 0.019	0.504*	-		
Pb	0.073	0.108	0.604*	0.447*	–	
Cr	− 0.312	− 0.275	0.317	0.357	0.517	–

*Zn* zinc, *Cu* copper, *Se* selenium, *Mn* manganese, *Pb* lead, *Cr* chromium.

***p < 0.001.

Table 5 presents a correlation analysis between the concentration ratios of selected heavy metals in umbilical cord blood and anthropometric variables in the mothers and newborns studied. We found a statistically significant positive correlation between the copper and zinc concentration ratio and the newborn’s head circumference (p = 0.031). There were no statistically significant correlations between the demographic and medical variables in mothers and newborns and lead/selenium and manganese/selenium concentration ratios in umbilical cord blood (p > 0.05).

**Table 5:** Correlations between concentration ratios of selected heavy metals and demographic and anthropometric data from mothers and newborns.

Parameters		Pb/Se	Mn/Se	Cu/Zn
		r		
Maternal data	Age of mothers	− 0.173	− 0.063	− 0.027
	Body weight before pregnancy	0.020	− 0.022	0.064
	Body growth	0.199	0.053	0.052
	Weight gain during pregnancy	0.029	0.067	0.048
Newborn data	Birth weight	0.207	− 0.065	0.124
	Birth lenght	0.119	− 0.129	− 0.026
	Head circumference	0.094	− 0.120	0.322*
	Chest circumference	0.198	− 0.180	0.072

*Pb* lead, *Se* selenium, *Mn* manganese, *Cu* copper, *Zn* zinc, *r* Pearson correlation coefficient.

*p < *0.05.*

## Discussion

The monitoring of human exposure to environmental chemicals is possible by means of analyzing concentrations of individual substances in biological samples^17^. The following metals were identified in the umbilical cord blood samples analyzed in the present study: lead, zinc, copper, manganese, selenium, chromium. The levels of other metals: cadmium, nickel, molybdenum, cobalt, antimony, thallium and vanadium were below LOD. This may point to low environmental exposure to these elements or high efficiency of the placental barrier among women from Lublin County. Studies conducted in regions of Poland that are more polluted than Lublin Province—in Lesser Poland and Silesia—demonstrated the presence of cadmium in umbilical cord blood, which was not observed in the present study^20–25^. In a study by Kiebała et al. on the content of chromium, copper, nickel, lead and zinc in street dust collected along the main traffic routes in Lublin, the highest concentration (of all the metals studied) was observed for zinc and the lowest for lead^23^. In the present study, zinc was the element with the highest concentration in the umbilical cord blood samples analyzed. The subject literature describes the relationship between zinc levels in umbilical cord blood and anthropometric parameters in newborns^12,25,26^. In a study by Kucukaydin et al., the level of zinc in umbilical cord blood in women experiencing preterm delivery with PROM (premature rupture of membranes) was 117.0 ± 43.0 μg/L and was lower than in the group of women giving birth without PROM (157.0 ± 45.0 μg/L)^27^. Likewise, Akdas et al. observed that the level of zinc in umbilical cord blood decreases in conditions complicating pregnancy as compared to physiological pregnancies^24^. When analyzing the concentrations of microelements and metals in umbilical cord blood among women in Poland, Gari et al. found that the median for zinc was 1000 μg/L^28^, which is a lower value than that observed in the present study. Cabrera-Rodriguez et al. did not observe any association between the content of zinc in umbilical cord blood and the birth weight of newborns^16^. These results are in line with our findings, which showed that zinc levels in umbilical cord blood were not significantly associated either with birth weight or with other anthropometric parameters in newborns.

Suliburska et al. observed a relationship between the content of copper in amniotic fluid and fetal biparietal diameter, abdominal circumference and femoral length^29^. In turn, our study showed that copper levels in umbilical cord blood samples correlated positively with the head circumference and negatively with the chest circumference of newborns. In their study on a Chinese population, Li et al. reported that copper content in umbilical cord blood was 123.1–699.6 μg/L (median: 298.2 μg/L)^30^. Our study found considerably higher copper levels in the samples analyzed than those in the above-mentioned paper. Lower copper levels in umbilical cord blood were also reported by Sakamoto et al. in a population of Japanese women (median: 438 µg/L)^31^. Copper levels observed by Elkabany et al. in Egypt were similar to those reported in our study^32^. Studies from Poland and abroad point to a relationship between the concentration of copper in umbilical cord blood and birth weight^12,22^. However, our results do not corroborate this association.

In their study on non-smoking women with uncomplicated pregnancies, Lewicka et al. observed that copper levels in amniotic fluid were more frequently reduced in overweight or obese patients than in women with a normal BMI^33^. In our study, there was a positive correlation between the copper content in umbilical cord blood and pregnancy weight gain in women living in Lublin County. A high blood concentration of copper together with a low level of zinc is associated with oxidative stress and development of cancers, including endometrial cancer^1^. We observed a very strong, positive correlation between the levels of zinc and copper in the umbilical cord blood samples studied. The statistical analysis revealed that the greater the disproportion between the levels of copper and zinc in umbilical cord blood, the greater the head circumference at birth.

The results of our study demonstrated a strong positive correlation between the level of selenium and the levels of lead and manganese. Selenium blood levels may depend on various factors, such as diet, supplementation, age, gender, geographic location or genes^34,35^. Furthermore, the selenium concentration in peripheral blood in women is associated with body weight and BMI^36^. We did not observe such associations when analyzing selenium levels in umbilical cord blood among female inhabitants of Lublin County. A study on healthy newborns conducted by Bebas et al. demonstrated a significantly higher selenium level in umbilical cord blood in newborns at term (72.25 ± 10.5 µg/L) as compared to infants born before 37 weeks of pregnancy (64.85 ± 7.67 µg/L)^37^. These values are nearly 50% lower than the selenium levels in our samples. Low selenium status is associated with lower activity of glutathione peroxidase (GPx), which enhances antioxidative protection of the placenta and indirectly influences fetal growth, which may lead to intrauterine growth restriction (IUGR)^38^. Yang et al. suggested that an elevated selenium level (≥ 63.1 µg/L) may inhibit the toxic effect of manganese on neurological development and that the lower the selenium concentration, the weaker its protective effect^39^. The interaction between the presence of selenium in umbilical cord blood and the level of manganese has also been confirmed in the present study. In a group of Japanese women, the mean selenium content in umbilical cord blood amounted to 139 µg/L (126–157 µg/L)^31^, which is slightly lower than the values observed in our study.

Röllin et al. demonstrated that the level of manganese in umbilical cord blood ranges between 25.8 μg/L and 43.0 μg/L, and higher levels of this element were found in women living in urban areas as compared to women living in rural areas^40^. This may be related to environmental exposure, as the level of manganese in atmospheric air is higher in urban areas with heavy traffic than in villages^41^. Vigeh et al. suggested that prenatal exposure to manganese may be associated with fetal growth. Higher manganese status in umbilical cord blood was observed in the case of IUGR (44.7 ± 19.1 μg/L) as compared to uncomplicated pregnancies (38.2 ± 13.1 μg/L)^42^. Lin et al. reported that the level of manganese in umbilical cord blood was 47.0 ± 1.4 μg/L. In a study by Guan et al., the mean manganese concentration in umbilical cord blood samples was 78.75 µg/L. Higher concentrations of this element were observed in pregnant women exposed to hazardous occupational factors. There was a statistical curvilinear relationship (inverted U shaped) between the concentration of manganese in umbilical cord blood and birth weight, head circumference and chest circumference^15^. Compared to all the studies cited above, our study did not confirm significant relationships between manganese levels in umbilical cord blood and anthropometric parameters in newborns.

Lin et al. found that the mean level of lead in umbilical cord blood was 12.9 ± 7.2 μg/L^43^. In a study conducted among women from Northeast Japan, Iwai-Shimada et al. reported that the lead level in umbilical cord blood was 8.02–12.5 μg/L (median: 9.89 μg/L)^44^. When analyzing umbilical cord blood taken directly after delivery from Spanish women following physiological pregnancy, Bocca et al. observed that the mean concentration of lead in the samples was 7.9 μg/L^2^. Our study showed higher lead concentrations in umbilical cord blood from female inhabitants of Lublin County, with the mean value of 26.25 μg/L. Based on her study among Polish women who gave birth vaginally after an uncomplicated pregnancy, Durska demonstrated that the mean lead level in umbilical cord blood was 21.4 μg/L. The lead content in umbilical cord blood did not have an influence on the health condition and anthropometric measurements in newborns, and was not associated with maternal education, occupation or social standing^45^. A study conducted in Poland by Gari et al. pointed to lower lead levels in umbilical cord blood than those observed in our study—median: 9.9 μg/L^28^. A study by Gundacker et al. found a negative correlation between the mean lead concentration in umbilical cord blood and pregnancy duration and birth weight. The median concentration of this element in umbilical cord blood was 13 μg/L^46^. Our study showed that the lead level in umbilical cord blood was lower than the cited value. Lin et al., who studied placental lead transport, reported that the mean lead concentration in umbilical cord blood was 12.9 ± 7.2 μg/L. Lower levels of this metal were observed in cases of higher zinc or manganese levels in maternal blood^43^. Our study showed a positive correlation between the lead content in umbilical cord blood and the levels of manganese and zinc. This correlation may suggest that the placental transfer of zinc and manganese is enhanced by high lead levels in pregnant women.

Exposure to chromium during pregnancy may be associated with fetal growth restriction. Peng et al. demonstrated that the higher the chromium concentration in urine samples from pregnant women, the smaller the maternal abdominal circumference and the estimated fetal weight assessed with ultrasound in the second and third trimesters^10^. Studies conducted by Huang et al. and Pan et al. among pregnant Chinese women showed that the mothers of preterm babies in a population environmentally exposed to chromium had higher urine chromium levels than women who gave birth at term^11,47^. Thus, chromium may be a toxic factor for fetal growth and a potential etiological factor for premature birth. In their study on the influence of cord blood elements on fetal development, Cabrera-Rodriguez et al. observed a significant relationship between the content of chromium in umbilical cord blood and body length at birth. The higher the concentration of this element, the shorter the body length^16^. Our study demonstrate a significant association between the level of chromium and head circumference in newborns. We observed higher chromium concentrations in newborns with a smaller head circumference. Cabrera-Rodriguez et al. showed that both individual element concentrations and the sum of chromium, nickel and antimony levels in umbilical cord blood may be a risk factor for low birth weight^16^.‬‬‬‬‬

The use of umbilical cord blood as a biomarker of fetal exposure to environmental pollution will increase the awareness of the potential health consequences for the child later in life, facilitate their identification and allow for a fast implementation of targeted therapy^48,49^.

### Strengths and limitations

The present study provides information on heavy metal accumulation in umbilical cord blood among female inhabitants of Poland, whose pregnancy occurred without complications. We analyzed relationships between element concentrations in umbilical cord blood samples and anthropometric and demographic data from mothers and newborns. The strength of our study is that the analysis focused on not only on the birth weight and length but also on the head and chest circumference of the newborns. The subject literature does not include many reports analyzing relationships between weight gain during pregnancy and heavy metal concentrations in umbilical cord blood.

The present study has certain limitations. First, the sample size was relatively small, which stemmed from the quite restrictive inclusion criteria. The largest group of women excluded from the study were patients with pregnancy-related comorbidities such as hypothyroidism, diabetes and hypertension. Furthermore, the women studied lived in a limited administrative area in Poland. The high-resolution inductively coupled plasma optical emission spectroscopy (ICP-OES) was the method used to measure heavy metals in umbilical cord blood. Considering the low detection levels for the analyzed heavy metals, perhaps other methods of analysis would be more sensitive. The seasonality of the umbilical cord blood samples taken was not analyzed either. Further research is needed to understand the impact of changes caused by heavy metals on mothers and their babies and to verify whether the level of heavy metals in umbilical cord blood can be used as a biomarker to determine the future health of exposed children.

## Conclusions

Accumulation of heavy metals in the body starts already at the fetal stage of life and its consequences can manifest not only during pregnancy and birth, but also in the neonatal period as lower body weight or length. The placenta is a filtering organ forming a protective barrier against heavy metal exposure^4,8^. In the present study, cadmium, molybdenum, cobalt, antimony, thallium and vanadium were not detected in umbilical cord blood samples taken from the study participants. Due to the relatively good quality of the participants’ living environment, the levels of these metals may have been marginal. Another explanation is that the metals were present in tissues but retained in the placenta. Differences in trace element concentrations in biological samples may stem from the geographic location, seasonality of material collection, environmental pollution, availability, preparation and processing of food, cultural practices and ethnic differences in body composition^2,7,16,33^.

Our analysis showed that weight gain in pregnant women is positively correlated with the copper level in umbilical cord blood. It has been established that BMI is positively correlated with copper concentration in peripheral blood. Excess copper is more often observed in obese patients^1,33^. These results may serve as the basis for determining reference values for this element in pregnant women. There is an association between head circumference at birth and the chromium concentration in umbilical cord blood: the greater the weight, the higher the lead content, and the greater the circumference, the lower the chromium content. Copper and zinc levels in umbilical cord blood are positively correlated with head circumference at birth. The higher the zinc level, the higher the copper level; the higher the manganese level, the higher the lead and selenium levels; and the higher the lead level, the higher the selenium and chromium levels.

The effects of toxic heavy metals, whose concentration is analyzed in umbilical cord blood samples, should be considered not only in the context of single measurements, but also that of metal mixtures emitted into the environment. Trace elements such as zinc, copper, manganese, selenium and chromium affect the normal course of metabolic, biochemical and enzymatic processes. Interactions between elements may have an impact on fetal homeostasis^31^. In turn, the placental transfer of toxic substances, e.g. lead or drugs, is facilitated by low levels of essential elements such as zinc, manganese and selenium in maternal blood^43^. The co-occurrence of various substances may change the bioavailability or toxicity of every single one of them. Measurements of individual elements in umbilical cord blood may be insufficient to determine the actual exposure to contaminants in pregnant women and fetuses, and subsequently newborns. Inorganic elements, both individually and in mixtures, are associated with lowered anthropometric parameters in newborns^16^.

It is necessary to undertake relevant activities monitoring the concentration of harmful chemicals in the environment. What is important is to raise social awareness of this risk and provide adequate health education to foster early prevention of excessive exposure to contaminants present in peoples’ natural living environment.

## Data Availability

The data presented in this study are available on request from the corresponding author.
